# Photodynamic Therapy with Tolonium Chloride and a Diode Laser (635 nm) in the Non-Surgical Management of Periodontitis: A Clinical Study

**DOI:** 10.3390/jcm12165270

**Published:** 2023-08-13

**Authors:** Marwan El Mobadder, Samir Nammour, Kinga Grzech-Leśniak

**Affiliations:** 1 Laser Laboratory, Oral Surgery Department, Wroclaw Medical University, 50-425 Wroclaw, Poland; kinga.grzech-lesniak@umw.edu.pl; 2 Department of Dental Sciences, Faculty of Medicine, University of Liege, 4000 Liege, Belgium; s.namour@uliege.be; 3 Department of Periodontics, School of Dentistry, Virginia Commonwealth University, Richmond, VA 23298-0566, USA

**Keywords:** periodontitis, periodontal disease, scaling and root planing, adjunctive therapy, photodynamic therapy, antimicrobial photodynamic therapy, disinfection, periodontal pockets

## Abstract

This study aimed to evaluate the efficacy of photodynamic therapy (PDT) using tolonium chloride and a 635 nm diode laser as an adjunct to non-surgical periodontitis treatment, specifically scaling and root planing (SRP) alone. A total of 32 patients with a pocket probing depth > 5 mm were included in the study. Among them, 16 patients underwent SRP alone (control group), and the remaining 16 patients received SRP along with PDT (study group). The PDT procedure utilized a 635 nm diode laser (Smart M, Lasotronix, Poland) and tolonium chloride. Clinical periodontal parameters, such as the plaque index (PI), bleeding on probing (BOP), gingival recession (GR), probing pocket depth (PPD), and clinical attachment loss (CAL), were assessed before treatment (T0) and at 3 months after treatment (T3). At T3, both groups demonstrated a significant reduction in the PI, BOP, PD, and CAL compared to T0. The SRP + PDT group displayed a significant reduction in PPD (3.79 mm ± 0.35) compared to the SRP alone group (4.85 mm ± 0.42) at T3. Furthermore, the SRP + PDT group exhibited a significant reduction in CAL (5.01 ± 0.81) compared to the SRP group (5.99 ± 1.08) at T3. Within the study’s limitations, it was concluded that PDT, with tolonium chloride and a 635 nm diode laser, significantly contributed to the non-surgical treatment of periodontitis.

## 1. Introduction

Periodontitis is a multifactorial, chronic inflammation characterized by the continuous destruction of the periodontal tissues, which provide support to the tooth [1,2,3]. Clinical manifestations of periodontitis include a loss of periodontal attachment, probing pocket formation, and gingival bleeding. Radiographically, evidence of vertical and/or horizontal alveolar bone loss is observed [4]. Due to its high prevalence and significant impact on patients’ quality of life, periodontitis has become a major public health concern [5,6]. It is notably associated with a substantial proportion of edentulism, mastication dysfunctions, and is considered a potential risk factor for various systemic diseases [5,6]. As per the guidelines provided by the European Federation of Periodontology (EFP), the current treatment of periodontitis comprises four consecutive steps: promoting good oral hygiene and a healthy lifestyle to reduce inflammation, performing thorough professional scaling and root planing, and if required, implementing more complex treatments, including surgical interventions. Finally, long-term supportive care is essential to prevent relapse [7]. Non-surgical mechanical debridement has limitations that can impede successful treatment [8,9]. Local factors, such as deep periodontal pockets (>5 mm), and anatomical challenges, like fissures, concavities, and furcation involvement, hinder effective debridement, leading to sub-optimal outcomes of periodontitis treatment [10,11,12]. To address these limitations associated with non-surgical mechanical debridement, additional approaches have been proposed to complement, but not replace, the standard of care [13,14,15,16,17,18]. These approaches primarily focus on enhancing the bactericidal effect and promoting periodontium repair. One such approach introduced in the management of periodontal diseases is photodynamic therapy (PDT) also referred to as antimicrobial photodynamic therapy [13,14,15,16,17,18].

PDT involves the utilization of a photosensitizer or photosensitizing agent, which is activated by light emitted from a laser or another light source [19,20,21,22]. This interaction between the drug and the photosensitizer ultimately results in the destruction of specific targeted cells or bacteria. In the field of medicine and dentistry, PDT offers a broad spectrum of advantages [23]. For instance, in odontology, PDT has demonstrated positive outcomes in treating pre-neoplastic lesions and leukoplakia of the oral cavity [24]. It has also shown promise in the preventive and curative management of oral complications arising from cancer treatment, such as oral mucositis [25,26], and treating oral candidiasis [27]. These are just a few of the indications that can be found in the existing literature. Considering that subgingival plaque removal (SPR) through contact with soft and hard tissues only offers limited debridement, incorporating PDT as an adjunctive tool could potentially augment the depth of disinfection. Nevertheless, to substantiate this claim, studies must present conclusive evidence showcasing the enhancement achieved with PDT in disinfection depth. This has been attributed to the deeper penetration of the solution and light, resulting in an increased eradication of pathogens and, consequently, better periodontium reparation. Recognizing these potential advantages, PDT has been proposed for the management of periodontitis [28]. Various photosensitizing agents and light sources can be employed in PDT. Despite the growing interest in PDT for periodontitis treatment, a thorough review of the literature revealed a lack of clinical or bacteriological studies evaluating the effectiveness of tolonium chloride as a photosensitizer when coupled with a 635 nm diode laser in PDT for non-surgical periodontitis treatment. Hence, there remains a gap in the existing research regarding this specific combination’s efficacy. This study aimed to assess the effectiveness of photodynamic therapy (PDT) using tolonium chloride and a 635 nm diode laser in the treatment of periodontal pockets with probing pocket depths of more than 5 mm. Our evaluation focused on various clinical periodontal parameters, including the plaque index (PI), bleeding on probing (BOP), gingival recession (GR), probing pocket depth (PPD), and clinical attachment loss (CAL). These parameters will be measured before the intervention and three months after the intervention. The null hypothesis for this research is that no significant differences will be observed in the clinical periodontal parameters between the conventional treatment group (SRP only) and the study group (SRP + PDT).

## 2. Materials and Methods

### 2.1. Design of the Study

This study included 32 patients, each of whom had a periodontal pocket depth greater than 5 mm. The patients were randomly divided into two groups: the control group and the study group. The control group, consisting of 16 patients (SRP group; n = 16), underwent non-surgical mechanical debridement for periodontitis in accordance with the guidelines provided by the European Federation of Periodontology (EFP). This control group consisted of 10 females and 6 males and had an average age of 43 years old. The study group also comprised 16 patients who received the same treatment as the control group but with the addition of photodynamic therapy (PDT). This study group consisted of 12 females and 4 males and presented an average age of 48 years old. The experiment involved using a 635 nm diode laser (Smart M, Lasotronix, Warsaw, Poland) and a photosensitizer solution of tolonium chloride (Lasotronix, Warsaw, Poland) (SRP + PDT group; n = 16). To assess the power of our study, we conducted a power calculation based on a commonly used effect size for medical studies (Cohen’s d = 0.5), an alpha level of 0.05 to control the risk of Type I errors, and a sample size of 16 patients in each group. Using these parameters, we estimated the power of our study to be approximately 0.67. 

The study was conducted following the principles outlined in the Declaration of Helsinki and was approved by the Bioethics Committee at the Medical University of Wroclaw under the reference number KB-28/2022. Prior to their enrollment in the study, all patients provided written informed consent.

### 2.2. Randomization 

In this study, randomization was employed to ensure an unbiased and equitable allocation of participants into two distinct groups. The process of randomization was carried out using Microsoft Excel, a widely used spreadsheet software. To achieve random assignment, a list of all eligible participants was created, and each participant was assigned a unique identifier. Then, a random number generator function within Excel was utilized to generate random numbers for each participant. The participants were subsequently sorted based on these random numbers in ascending order. The first half of the sorted list was allocated to scaling and root planing only, while the second half formed the group SRP + photodynamic therapy with 635 nm diode laser. This approach ensured that each participant had an equal chance of being placed in either group, minimizing the potential for selection bias, and enhancing the validity of the study’s findings. Through the use of Microsoft Excel for randomization, this process was made efficient and transparent, allowing for a robust and reliable investigation of the research question.

### 2.3. Participants

#### 2.3.1. Criteria of Inclusion

- Patients with PPD > 5 mm (stage III or IV based on the EFP and AAP’s classification).

#### 2.3.2. Criteria of Exclusion

- Mobility on the concerned tooth.
- Presence of any systemic disease that contradicts a non-surgical periodontal treatment.
- Uncontrolled diabetes.
- Patients with a plaque index > 30%.
- Patients under antibiotics or probiotics or any other adjunction within the last six months.
- Patients under immunosuppressants within the last 6 months.
- Heavy smokers (>10 cigarettes/day).
- Lactating and pregnant woman.

### 2.4. Treatment Protocol for the SRP Group

Patients that were allocated to the SRP group received proper oral hygiene instructions, which included guidance on adopting an adequate tooth brushing technique and using interdental brushes for interdental cleaning. Following this, professional subgingival plaque removal (SRP) was performed using an ultrasonic piezoelectric scaler (Piezosteril 6, Castellini, Cazzago San Martino, Italy) for the entire mouth. Additionally, instrumentation with curettes (Universal and Gracey curettes) was conducted. Subsequently, a chlorhexidine 0.12% solution (Eludril pro mouthwash, Pierre Fabre Oral Care, Paris, France) was used to irrigate the sulcus for an average duration of 10 s. This described treatment protocol was the only treatment administered for the conventional group.

### 2.5. Treatment Protocol for the SRP + Photodynamic therapy Group (SRP + PDT)

Patients included in the SRP + PDT group received the same conventional treatment protocol as described in Section 2.3. After the initial conventional treatment, photodynamic therapy (PDT) was specifically performed for the periodontal pockets of the 16 patients with probing pocket depths (PPD) greater than 5 mm. The PDT protocol began with irrigating the targeted pocket using tolonium chloride. This was achieved using a dedicated irrigation tip, which was placed at the depth of the pocket (PAD smart solution, Lasotronix, Warsaw, Poland). The irrigation process was performed slowly and stopped once the tolonium chloride solution was observed at the free gingival margin of the pocket. Subsequently, a 30 s waiting period was observed. Following the waiting period, a specialized applicator for photodynamic therapy (perio applicator, Lasotronix, Warsaw, Poland) was introduced into the pocket to the appropriate depth, and the laser irradiation was initiated. During the irradiation process, the tip was moved in a steady vertical inward and outward motion while progressing slowly at an average rate of 1 mm per second. This movement was executed to ensure that the entire pocket was adequately covered. The irradiation parameters were as follows: 200 mW, 30 s, energy density of 6 J/cm^2^, tip diameter of 400 µm, and using contact and continuous modes of irradiation. This precise irradiation protocol was then repeated two more times for the same targeted periodontal pocket during the same treatment session, resulting in a total of three repetitions.

### 2.6. Clinical Assessment and Follow-Up

A single calibrated examiner (M.E.M) noted the values obtained of the clinical parameters for both groups before intervention (T0) and at three months after intervention (T3). The PI, BOP, GR, PPD, and CAL were calculated prior to intervention (T0) and at 3 months post-intervention (T3). 

#### 2.6.1. The Plaque Index (PI)

The PI was calculated through scoring from 0 to 3 on four surfaces of the following teeth: #16, #12, #24, #36, #32, and #44, following which the mean value of the obtained scores was noted. 

#### 2.6.2. Bleeding on Probing (BOP)

BOP was calculated with a periodontal probe (PCP UNC 15, HuFriedy, Chicago, IL, USA) on 6 sites for each tooth and the scores were noted in percentage (%) of gingival units that bled compared to the total number of units. 

#### 2.6.3. Gingival Recession (GR) and Pocket Depth (PD)

GR and PD was calculated with a periodontal probe (PCP UNC 15, HuFriedy, Chicago, IL, USA), were only assessed on the concerned periodontal pocket, and were measured in mm.

#### 2.6.4. Clinical Attachment Loss (CAL)

Clinical attachment loss was assessed by adding the PPD value to the gingival recession value if GR was present and by subtracting the distance from the CEJ to the gingival margin level from the PPD when the gingival margin was coronal to the cemento-enamel junction (CEJ). 

### 2.7. Statistical Analysis

Statistical analysis was performed using Sigma five^®^ software (GraphPad Prism 5, San Diego, CA, USA), and a *p*-value of less than 0.05 was considered statistically significant. Mean values and standard deviations (STDs) were calculated for all parameters in both the control and SRP + PDT groups at two time points: prior to the intervention (T0) and three months after the intervention (T3). To determine the normality of the data, Kolmogorov–Smirnov tests were used. The one-way ANOVA, coupled with a Newman–Keuls multiple comparison test as a post hoc test, was used for the assessment of statistically significant differences within groups at different follow-up times and between the two groups. This statistical analysis allowed for the comparison of data at different time points and between the control and SRP + PDT groups to ascertain any significant differences.

## 3. Results

### 3.1. Plaque Index and Bleeding on Probing

There was a significant reduction in the mean values of the plaque index in both groups at three months compared to their respective values at T0. After three months, the mean values were 1.35 ± 0.54 and 1.38 ± 0.37 for the SRP and SRP + PDT groups, respectively; however, no statistically significant difference was found between these groups when compared together. BOP significantly decreased after treatment for both groups with no significant difference observed between both groups (Table 1).

### 3.2. Gingival Recession

Following the intervention (T3), both groups exhibited a significant increase in mean gingival recession compared to the baseline measurement at T0. GR was 1.14 ± 0.53 mm and 1.22 ± 0.46 mm for the control and study groups, respectively. At T3, no significant difference in the mean values of GR was noted between both groups (Table 1). 

### 3.3. Probing Pocket Depth

Mean values of the PPD decreased significantly in both groups from T0 to T3. At three months of follow-up, PPD values dropped from 7.11 ± 0.73 mm (T0) to 4.85 ± 0.42 mm for the control group, and from 6.69 ± 0.83 mm to 3.79 ± 0.35 mm for the study group, respectively. Within groups, a significant reduction in the mean PPD values was obtained for the SRP + PDT group compared to the control group (Table 1).

### 3.4. Clinical Attachment Loss

In both groups, a significant reduction in CAL was obtained between T3 and T0. Values for the control group dropped from 7.82 ± 1.08 mm (T0) to 5.99 ± 1.08; for the study group, values dropped from 7.38 ± at T0 to 5.01 ± 0.81. When both groups were compared at T3, a statistically significant difference was noted (Table 1).

## 4. Discussion

In this study, the supplementary application of photodynamic therapy (PDT) using a 635 nm diode laser and tolonium chloride demonstrated a significant enhancement in the efficacy of non-surgical treatment for periodontitis. These results affirm that PDT, when combined with scaling and root planing (SRP) within our specific protocol, leads to a more profound disinfection of the periodontal pockets. Hence, the null hypothesis was rejected. This effect appears to be associated with a more extensive reduction of bacteria, their toxic by-products, like lipopolysaccharides, and the neutralization of host pro-inflammatory cytokines. The ability of PDT to achieve a deeper reduction of bacteria is likely attributed to the profound penetration of the photosensitizer within the periodontal tissues when activated with the 635 nm wavelength light. This enables an extensive bactericidal effect on the bacteria residing not only in the deep periodontal tissues but also on the non-periodontal mucosal surfaces. Previous studies have shown the presence of periodontopathogens, such as *Aggregatibacter actinomycetemcomitans*, in the depth of epithelial cells within periodontal pockets and outside the gingival tissues [29,30]. It seems that conventional mechanical debridement could be enhanced with PDT, which enables the disinfectant to penetrate the depth of the periodontal tissues due to the nature of the light (with deep tissue penetration capabilities) and the liquid solution used; therefore, this might have resulted in the achievement of a more comprehensive and profound disinfection [26,27,28]. This can suggest that PDT might be a valuable adjunct to conventional therapy, providing an enhanced and targeted approach for the treatment of periodontitis. However, further bacteriological investigations in future studies must confirm this hypothesis. Our suggested protocol showed a significant reduction in the mean values of PPD and clinical attachment loss (CAL) when compared to SRP alone; this suggests that PDT seems to have a better reparation of the periodontium attributed to the fact that an enhancement of the disinfection of the periodontal tissues will lead to a better reparation of the periodontium. Additionally, the calculation of the plaque index was statistically the same in both the control and study groups, suggesting that both groups had a good control of oral hygiene and followed the instructions that were given during the first session.

To comprehensively interpret the results of our study, it is crucial to delve into the mechanism of action of photodynamic therapy (PDT). Initially, PDT involves the activation of a photosensitizer agent from its ground state to a highly energized triplet state through irradiation with a specific wavelength [23,31,32]. This excited photosensitizer, possessing a longer life-time compared to ordinary reactive oxygen species (ROS), interacts with the surrounding molecules. In its triplet state, a cascade of reactions occur, leading to the generation of cytotoxic species [33,34]. Two distinct pathways have been proposed for the reactions of the triplet state: Type I and Type II [23]. The Type I reaction centers on hydrogen atom abstraction between the stimulated state of the photosensitizer and the organic substrate molecule within the cells. This process produces highly reactive free radicals and radical ions [23]. These free-radical species further interact with endogenous molecular oxygen, resulting in the production of highly reactive oxygen species, including superoxide, hydroxyl radicals, and hydrogen peroxide. These reactive oxygen species essentially “attack” the cell membrane, causing its disintegration and leading to irreversible biological damage [23,33,34]. On the other hand, Type II reaction involves the direct interaction of the triplet state photosensitizer with molecular oxygen. This interaction leads to the production of an electronically excited and highly reactive state of oxygen called singlet oxygen (O_2_). Singlet oxygen exhibits high chemical reactivity and reacts with various biological structures, resulting in oxidative damage and lethal actions on bacterial cells by disrupting their cell membranes and walls. Consequently, singlet oxygen possesses the capability to eliminate viruses, bacteria, protozoa, and fungi. In biological systems, singlet oxygen has a lifetime of 0.04 µs and a radius of action of 0.02 µm [23]. By understanding the underlying mechanism of action of PDT, we can gain insights into how it exerts its potent antimicrobial effects, providing a rationale for its effectiveness as an adjunct therapy in the non-surgical treatment of periodontitis.

Numerous studies in the literature have explored the use of photodynamic therapy (PDT) for managing deep pockets in periodontitis [35]. For instance, a systematic review and meta-analysis (SR–MA) investigating the impact of adjunctive PDT on clinical attachment loss (CAL) in stages II-IV grade C periodontitis was conducted [36]. This SR–MA [36] revealed that the use of PDT resulted in a significant improvement in CAL for patients with initial CAL measurements > 7 mm. However, no significant improvement was observed when CAL was initially less than 7 mm [36]. Nonetheless, certain aspects, such as the duration of irradiation, choice of photosensitizer, and standardization of parameters, require further establishment. Furthermore, Ramanauskaite et al. [37] published a systematic review and network meta-analysis on PDT in periodontal maintenance. The conclusions drawn by Ramanauskaite et al. highlighted that both single and multiple additional applications of PDT following SRP led to a notable reduction in bleeding on probing (BOP) compared to scaling and root planing alone. Interestingly, their study indicated that multiple applications of PDT did not yield superior results when compared to single applications [37]. Additionally, PDT was found to be effective in increasing clinical attachment loss when associated with SRP in the treatment of residual pockets in diabetic patients within a three-month follow-up period [37]. These studies underscore the potential benefits of PDT as an adjunctive therapy in the management of periodontitis, particularly in cases with deep pockets and challenging residual pockets in diabetic patients. However, standardization of various parameters and further research will be essential to optimize the clinical outcomes and fully exploit the advantages of PDT in periodontal therapy.

Several other adjunctive approaches have been suggested in the literature. El Mobadder et al. [38] found, in a microbiological study, that the TBC of pathogens inside deep pockets (>5 mm) decreases significantly following 3% H_2_O_2_ irrigation coupled with a diode laser (980 nm) [38]. However, their study was only bacteriological and with an absence of assessment on the count of the periodontopathogens. Additionally, El Mobadder et al. [39] revealed, in another study, that 0.5% sodium hypochlorite coupled with a 980 nm diode laser can result in a significant reduction in periodontal pocket depth, clinical attachment loss, and the count of the main periodontopathogens when compared to SRP alone [39]. However, a greater gingival recession was observed in the suggested protocol compared to SRP alone, which can be considered as a side effect of the treatment [39]. In contrast, in this study, the suggested protocol did not show any additional periodontal recession compared to SRP alone. Considering that PDT adds to the overall cost of periodontitis treatment, it may be prudent to consider its use as a viable option primarily for pockets greater than 6 mm. By focusing on cases where its potential benefits in improving treatment outcomes are likely to be most significant, patients and clinicians can make informed decisions to optimize treatment efficacy and cost-effectiveness.

This study presents several limitations. For example, although this study assessed the effect of the suggested protocol on the clinical periodontal parameters, the assessment of the periodontopathogens using either polymerase chain reaction (PCR) or total bacterial counts (TBCs) is absent. Moreover, a six-month follow-up after treatment could have been further accurate on the evolution of the overall clinical periodontal parameters. Furthermore, it is important to note that the sample size in this study can be considered relatively small. Hence, extended follow-up duration holds the potential to reinforce the robustness of our findings, thereby enhancing the confirmation of our results. Also, it is intriguing to evaluate the effect of photodynamic therapy (PDT) on cases of periodontitis characterized by associated vertical bone loss and angular morphological defects. This assessment aims to determine its impact on bone regeneration.

Although there is a considerable amount of evidence for the effectiveness of PDT, the clinical practice guidelines of the European Federation of Periodontology (EFP) does not support PDT in patients with periodontitis [7]. The EFP’s conclusion was based on findings from five randomized clinical trials involving a total of 121 patients, with an average follow-up period of six months and a single session of application. Regarding the American Academy of Periodontology, their review indicated that combining SRP (scaling and root planing) with PDT is recommended for treating aggressive periodontitis with a moderate level of certainty. However, due to the limitations of the available evidence, an accurate assessment of the clinical significance of the findings remains inconclusive. [40]. Thus, this clinical study can serve as a foundational stepping stone for future explorations along this trajectory.

## 5. Conclusions

Photodynamic therapy with tolonium chloride as a photosensitizer along with a 635 nm diode laser as a light source results in a significant improvement of the clinical periodontal parameters compared to scaling and root planing alone. However, further investigations are required. 

## Figures and Tables

**Table 1:** Representation of the clinical results obtained for both groups and at different times of follow-up.

Variable	SRP Only (n = 16)	SRP + PDT (n = 16)
Plaque index		
T0	2.11 ± 0.74 ^a^	2.17 ± 0.55 ^a^
T3	1.35 ± 0.54 ^b^	1.38 ± 0.37 ^b^
BOP		
T0	52.63 ± 12.14 ^a^	48.82 ± 13.5 ^a^
T3	16.32 ± 8.13 ^b^	17.87 ± 8.13 ^b^
GR		
T0	0.71 ± 0.35 ^a^	0.69 ± 0.27 ^a^
T3	1.14 ± 0.53 ^b^	1.24 + 0.46 ^b^
PPD		
T0	7.11 ± 0.73 ^a^	6.69 ± 0.83 ^a^
T3	4.85 ± 0.42 ^b^	3.79 ± 0.35 ^c^
CAL (mm)		
T0	7.82 ±1.08 ^a^	7.38 ±1.1 ^a^
T3	5.99 ±1.08 ^b^	5.01 ±0.81 ^c^

Identical superscripts indicate an absence of a statistically significant difference. The difference in superscripts indicates a statistically significant difference. *p*-value < 0.05; mm—millimeters.

## Data Availability

Present with the corresponding author and can be delivered upon reasonable request.
